# The triterpenoid saponin content difference is associated with the two type oxidosqualene cyclase gene copy numbers of *Pulsatilla chinensis* and *Pulsatilla cernua*

**DOI:** 10.3389/fpls.2023.1144738

**Published:** 2023-02-23

**Authors:** Xianju Liu, Yudi Xu, Jipeng Di, An Liu, Jinzhu Jiang

**Affiliations:** Key Laboratory of Beijing for Identification and Safety Evaluation of Chinese Medicine, Institute of Chinese Materia Medica, China Academy of Chinese Medical Sciences, Beijing, China

**Keywords:** triterpenoid saponin, *Pulsatilla chinensis*, *Pulsatilla cernua*, oxidosqualene cyclase (OSC), gene copy number

## Abstract

*Pulsatilla chinensis* is an important medicinal herb, its dried radix is used to treat the inflammation since ancient China. Triterpenoid saponins are proved to be the main active compounds of *Pulsatilla* genus. The triterpenoid saponin contents vary widely in different *Pulsatilla* species. But no enzyme involved in the triterpenoid saponin biosynthetic pathway was identified in *Pulsitilla* genus. This seriously limits the explanation of the triterpene content difference of *Pulsatilla* species. In this article, we obtained two oxidosqualene cyclase (*OSC*) genes from *P. chinensis* and *P. cernua* by touchdown PCR and anchored PCR. These two OSCs converted 2,3-oxidosqualene into different triterpenoids. The OSC from *P. cernua* is a monofunctional enzyme for *β*-amyrin synthesis, while the OSC from *P. chinensis* is a multifunctional enzyme for lupeol and *β*-amyrin synthesis, and the lupeol is the main product. Then we identified the 260th amino acid residue was the key site for the product difference by gene fusion and site-directed mutant technology. When the 260th amino acid residue was tryptophan (W260) and phenylalanine (F260), the main catalysate was *β*-amyrin and lupeol, respectively. Then we found that the expression of these two genes was strongly correlated with the lupeol-type and *β*-amyrin-type triterpenoid contents in *P. cernua* and *P. chinensis*. Finally, we found the gene copy number difference of these two genotypes leaded to the triterpenoid diversity in *P. cernua* and *P. chinensis*. This study provides useful information for the molecular breeding and quality improvement of *P. chinensis* and a molecular marker to identify the *P. chinensis* decoction pieces.

## 1 Introduction

*Pulsatilla chinensis* is a species of medicinal and ornamental plant, which belongs to Ranunculaceae, *Pulsatilla* genus. In China, the dried radix of *P. chinensis*, which is known as ‘Bai Tou Weng′, is a kind of traditional Chinese medicinal material (TCM), which has been proved could effectively treat dysmenorrhoea, testicular inflammation, and other inflammation in China and other countries from the 19^th^ century up to now (Lydston, 1882; Coley, 1922; Friese et al., 1996; Misra et al., 2021; Niu et al., 2023). The Chinese Pharmacopoeia defines anemoside B4 as the index component of *P. chinensis*, and the content of anemoside B4 in decoction pieces must be more than 4.6% (dry weight). There are more than 60 species in *Pulsatilla* genus (Misra et al., 2021). Because of the appearance similarity, the dried radix of *P. cernua* were usually sold as *P. chinensis* in the Chinese herbal medicine market, but there were few anemoside B4 in them.

**Figure 1:**
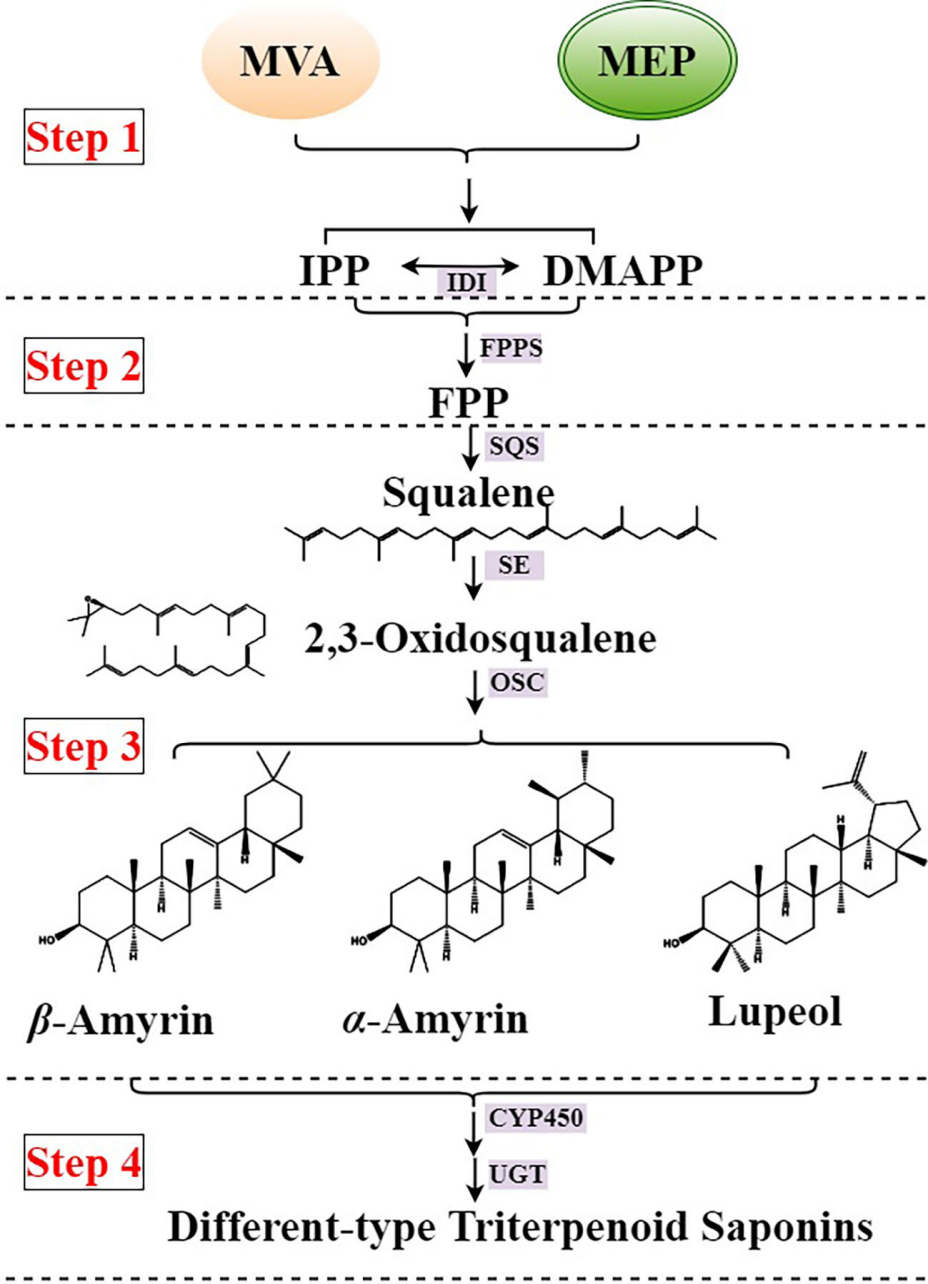
Biosynthesis of triterpenoid saponins in plants. IDI, IPP/DMAPP isomerase; FPPF, farnesyl pyrophosphate synthase; SQS, squalene synthase; SE, squalene epoxidase; OSC, oxidosqualene cyclases; CYP450, cytochromes P450; UGT, UDP-dependent glycosyltransferases.

Triterpenoid saponins are the main active compounds of the *Pulsatilla* genus, which show high biological activity in anticancer (Xu et al., 2012), neuroactive (Yoo et al., 2008), neuroprotective (Liu et al., 2012), antioxidant (Li et al., 2008), antimicrobial (Lee et al., 2001), and cytotoxic agents (Xu et al., 2013). There are abundant triterpenoids, especially lupeol-type and *β*-amyrin-type triterpenoid saponins in the roots of the *Pulsatilla* genus (Laska et al., 2019). The lupeol-type triterpenoid saponins abundantly accumulate in the roots of *P. chinensis*, and the *β*-amyrin-type triterpenoid saponins abundantly accumulate in the roots of *P. cernua*. The triterpenoid saponin type difference between *P. chinensis* and *P. cernua* make the source and application of decoction pieces more complicated, so clarifying the biosynthesis difference of triterpenoid saponins between *P. chinensis* and *P. cernua* is urgent. The main biosynthesis of triterpenoid saponins in plants is clear, and could be divided into four steps (**Figure 1**). The First step is the synthesis of terpenoid basic units isopentenylpyrophosphate (IPP) and dimethylallypyrophosphate (DMAPP). They were synthesized in two compartmentally separated metabolic pathways, mevalonate (MVA) pathway in the cytoplasm and methylerythritol phosphate (MEP) pathway in the plastid, and these two C5 precursors could interconvert into each other by IPP/DMAPP isomerase (IDI). The second step is that two IPP and one DMAPP units combine to form farnesyl diphosphate (FPP). The third step is that two FPP units synthesize squalene by squalene synthase (SQS), then squalene oxidized into 2,3-oxidesqualene by squalene epoxidase (SE). As the common precursor of triterpenoid and sterol, 2,3-oxidesequalene turned into lupeol, *β*-amyrin, *α*-amyrin, and other triterpenoids by oxidosqualene cyclases (OSC) (Krokida et al., 2013; Sun et al., 2013; Wang et al., 2021). The final step is triterpenoids were modified by cytochromes P450 (CYP450) and UDP-dependent glycosyltransferases (UGT) (Seki et al., 2015; Christ et al., 2019; Rahimi et al., 2019). However, no enzyme involved in the triterpenoid saponin biosynthetic pathway was identified in the *Pulsitilla* genus.

As the first enzyme caused triterpenoid saponin diversity, OSCs have received much attention since the 19th century. Only one OSC member is responsible for the synthesis of sterol in animals and fungi. While OSC always exists as gene family in plants. For example, there were 13 members in *Arabidopsis thaliana*, 16 members in *Tripterygium wilfordii*, and 9 members in *Momordica charantia* (Husselstein-Muller. et al., 2001; Han et al., 2021; Liu et al., 2021). Until 2022, there were more than 170 OSCs were obtained from 61 plant species (Chen et al., 2021; Wang et al., 2022). The catalysates of plant OSCs include a few monocyclic, bicyclic, tricyclic triterpene alcohols and many tetracyclic and pentacyclic triterpene alcohols (Chen et al., 2021; Wang et al., 2021; Lertphadungkit et al., 2022). In addition, site-directed mutagenesis results of some *OSC* genes indicate that a single key amino acid mutation is important to its product diversity. As concluded by Chen, et al., key amino acids, which important to the activity of OSC, were divided into 6 groups: Y118, Y410, M(W/L)C(Y/H)CR motif, DCTAE motif, and adjacent residues, C-terminus, and other residues (Chen et al., 2021). These results provide useful information to reveal the triterpenoid saponin biosynthesis difference between *P. chinensis* and *P. cernua.*

To explain the triterpenoid saponin biosynthesis difference between *P. chinensis* and *P. cernua*, in this study we obtained two *OSC* full-length CDS sequences, one from *P. chinensis* (*PchAS*) and the other one from *P. cernua* (*PcAS*) by touchdown PCR and anchored PCR. There are 27 differential amino acids between them. Then we analyzed the function of these two enzymes. Interestingly, the two OSCs convert 2,3-oxidosqualene into different triterpenoids. *PcAS* was a monofunctional enzyme for *β*-amyrin synthesis, while *PchAS* was a multifunctional enzyme for lupeol and *β*-amyrin synthesis, and lupeol was the main product. To find the key amino acid for the product diversity, fusion genes of *PcAS* and *PchAS* and site-directed mutant of *PcAS* were expressed in yeast. The final result shows that W260 (tryptophan) is important for *β*-amyrin synthesis, if this amino acid was mutated to F (phenylalanine), the products changed to lupeol and *β*-amyrin. Then we found the expressions of two genes were strongly correlated with the lupeol-type and *β*-amyrin-type triterpenoid content in *P. cernua* and *P. chinensis*. Finally, we analyzed the gene copy numbers by qPCR, and the result showed that the gene copy numbers lead to triterpenoid diversity of *P. cernua* and *P. chinensis*. This study provides useful information for the quality improvement of *P. chinensis* and a molecular marker to identify the *P. chinensis* decoction pieces.

## 2 Results

### 2.1 Cloning of putative lupeol and *β*-amyrin synthase gene from the roots of *Pulsatilla chinensis* and *Pulsatilla cernua*

**Figure 2:**
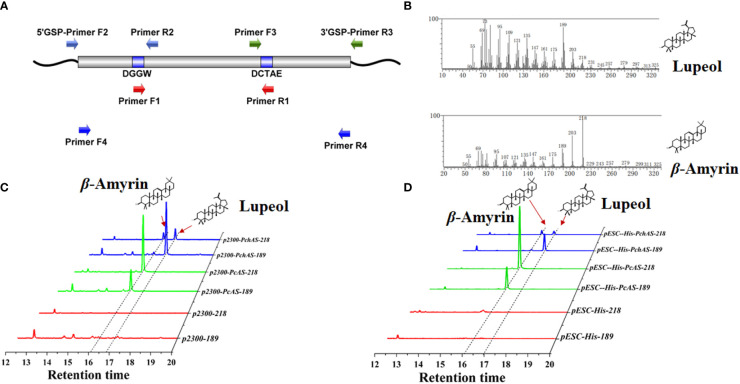
Functional characterization of *AS* homolog genes from *Pulsatilla ceruna* and *P. chinensis*. **(A)** A simplifed overview of gene cloning of *AS* from *P. ceruna* and *P. chinensis*; **(B)** Mass spectra of *β*-amyrin and lupeol detected by GC–MS; **(C)** GC–MS analysis of extracts from transient transformation *Nicotiana benthamiana* leaves which expressed *AS* genes from *P. cernua* (*PcAS*) and *P. chinensis* (*PchAS*). pCAMBIA2300 empty vector and pSAK277-*NbHAMGR* co-transformation *N. benthamiana* leaves were used as control; **(D)** GC–MS analysis of extracts from engineered yeast which expressed *PcAS* and *PchAS*. ERG7-deficient yeast that transformed with pESC-His empty vector was used as control; The 218 and 189 ion counts of *β*-amyrin and lupeol were shown, respectively.

To isolate *β*-amyrin and lupeol synthase genes, we obtained the full-length sequence of *OSCs* from *P. chinensis* and *P. cernua* by 3 kinds of PCR (**Figure 2A**). Firstly, we used the degenerate primers (IS1F, IS2R, TAS1F, and TAS1R, **Supplementary Table S1**) obtained from the previous study (Guhling et al., 2006). Degenerate PCR was performed by using cDNA from roots of *P. chinensis* and *P. cernua* as templates. Unfortunately, we didn′t obtain any products. Then we designed codehop primers (NEDG-F 8X, DQDH-R 16X, **Supplementary Table S1**), which with hybrid structure (5*′* consensus and 3*′* degenerate). Luckily, we got approximately 890 bp products from both *P. chinensis* and *P. cernua* (**Supplementary Figure S1A**). And this is corresponding to the distance between the codehop primers in the *β*-amyrin and lupeol synthase genes from the other plants. Then the DNA fragments were subcloned into the zero-background T-vector. 20 independent clones were selected to sequence. When blasted in NCBI database, all the sequences were similar to *Aralia elata β*-amyrin synthase (Wang et al., 2022), so in this article we named the *OSC* genes from *P. chinensis* and *P. cernua* as *PchAS* and *PcAS*, respectively. To obtain the full-length cDNA sequence of *PchAS* and *PcAS*, we used 5*′*- and 3*′*-RACE technology (because of the high sequence similarity, in this experiment we only used the core fragment of *PchAS*). We designed two independent primers (5*′*-GSP-R2-1/R2-2, 3*′*-GSP-F3-1/F3-2) for each flank and the primer sequences were listed in **Supplementary Table S1**. The PCR using both of these 5*′*-GSP primers and 3*′*-GSP primers could obtain products (**Supplementary Figure 1B**), and the products were subcloned into the T vector and sequenced. The *PchAS* cDNA contained an ORF of 2280 bp encoding 759 amino acid residues. At last, we obtained full-length sequences of *AS* genes from *P. chinensis* and *P. cernua* cDNA by Primer F4 and Primer R4 (**Supplementary Table S1**, **Supplementary Figure 1C**). The deduced amino acid sequences of PcAS and PchAS showed the highest identity of 86.69% and 85.64% to the *Nigella sativa β*-amyrin synthase NsbAS1 (Scholz et al., 2009), and both of them have 6 QXXXXXW, 1 MXCYCR, and 1 DCTAE domain, which were the conserved domains for OSC protein sequence. There are 27 different amino acid residues between PcAS and PchAS (**Supplementary Figures S2**, **S3**).

### 2.2 Functional analysis of the *PcAS* and *PchAS* genes in yeast and *Nicotiana benthamiana*

In order to elucidate the function of *PcAS* and *PchAS*, complete ORFs of them were amplified from root cDNAs using high-fidelity DNA polymerase and gene-specific oligonucleotides and inserted into yeast expression vector pESC-His by homologous recombination technology (AS-pESC-F/R primers for yeast expression vector were listed in **Supplementary Table S1**). In this vector, *PcAS* and *PchAS* genes were driven by the inducible promoter GAL10. Next, we expressed *PchAS* and *PcAS* in the lanosterol-deficient yeast strain ATCC40029, which could provide sufficient substrate for PcAS and PchAS. The fragment ion peak of lupeol and *β*-amyrin standards were shown in **Figure 2B** and **Supplementary Figure S4**. As shown in **Figure 2D**, PcAS produced *β*-amyrin as the single product (fragment ion peaks and retention time were the same as *β*-amyrin standard), while PchAS is a multifunctional enzyme, which produced lupeol and *β*-amyrin (fragment ion peaks and retention time were same as *β*-amyrin and lupeol standards). The main product of PchAS was lupeol, and *β*-amyrin was the secondary product (**Supplementary Figure S4**).

With the same method, we constructed the plant expression vectors, in which target genes *PcAS* or *PchAS* were driven by the constitutive promoter CaMV35S. In addition, we constructed a plant expression vector that constitutively overexpress *NbHMGR* gene (pSAK277-*NbHMGR*) to increase the substrate concentration of PcAS and PchAS. Then, these two genes were co-expressed with *NbHMGR* in *N. benthamiana* leaves through Agrobacterium-mediated transient transformation. The results showed that the gene functions in plants were the same as in yeast. That is, *PcAS* co-expressed with *NbHMGR* in *N. benthamiana* leaves produced *β*-amyrin as the single product. Similarly, *PchAS* co-expressed with *NbHMGR* in *N. benthamiana* leaves produced dominated lupeol and some *β*-amyrin (**Figure 2C** and **Supplementary Figure S4**).

### 2.3 Identifying the region leading to gene functional diversity by gene fusion

**Figure 3:**
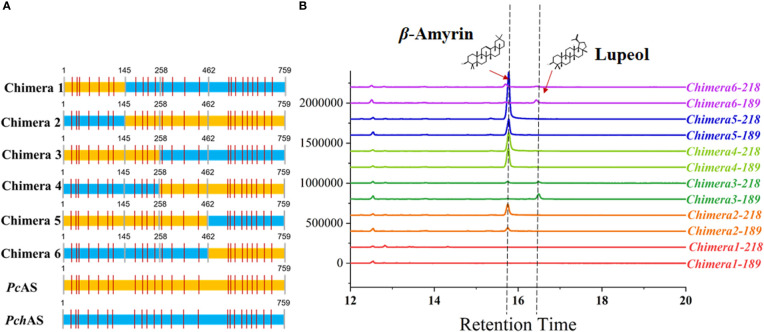
Functional characterization of chimera protein. **(A)** Diagram of the chimera protein. The colors indicate that fragment source: *P. chinensis* (blue) and *P. cernua* (yellow). **(B)** GC–MS analysis of chimera protein expressed yeast extraction. ERG7-deficient yeast that transformed with pESC-His empty vector was used as control. The 218 and 189 ion counts of *β*-amyrin and lupeol are shown, respectively. *PcAS* represent *AS* gene cloned from *P. cernua*, *PchAS* represent *AS* gene cloned from *P. chinensis*.

To identify the key regions that lead to the functional diversity between *PcAS* and *PchAS*, we separated each of these two genes into 3 fragments, fragment 1: 1-145 amino acid residues, fragment 2: 146-462 amino acid residues, and fragment 3: 462-756 amino acid residues (**Figure 3A**). Then we fused the different fragments from *PcAS* and *PchAS* and obtained six chimera genes by bridge PCR (**Figure 3A**). As shown in **Figure 3B**, chimera protein 3 and chimera protein 6 produced lupeol and *β*-amyrin in yeast (**Figure 3B**). However, chimera proteins 2, 4, and 5 only produced *β*-amyrin as the single product in yeast. In other words, when fragment 2 of the chimera protein is from *P. chinensis*, the chimera protein will show the characteristics of PchAS, which is the multifunctional enzyme, to produce lupeol and *β*-amyrin. When fragment 2 is from *P. cernua*, it will show the characteristics of PcAS, which is a monofunctional enzyme, to produce *β*-amyrin (**Figure 3B**). Therefore, fragment 2 is the key region for the *OSC* gene functional diversity between *P. chinensis* and *P. cernua*.

### 2.4 Identifying the key amino acids of PcAS and PchAS by site-directed mutagenesis

**Figure 4:**
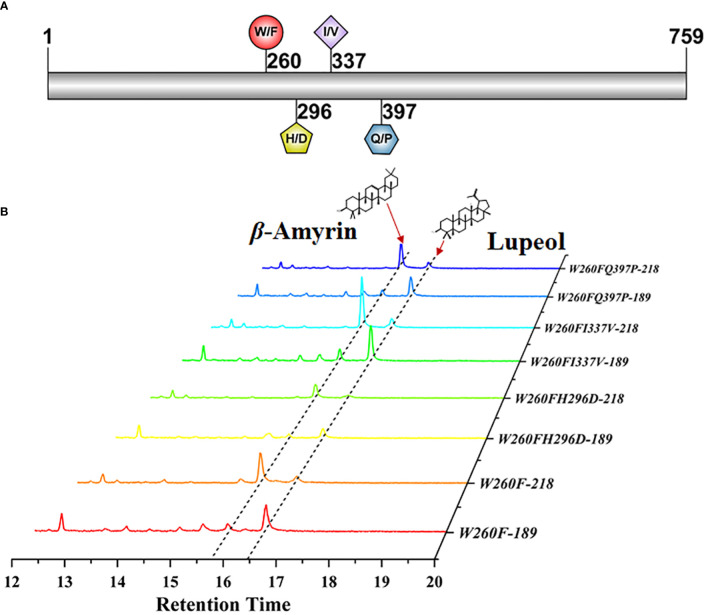
Functional characterization of the directional mutant protein W260F, W260FI337V, W260FH296D, and W260FQ397P. **(A)** Diagram of the mutant sites of *PcAS*. **(B)** GC–MS analysis of mutant protein expressed yeast extraction. The 218 and 189 ion counts of *β*-amyrin and lupeol are shown, respectively.

To further identify the key amino acid that led to the product difference between PchAS and PcAS, we directionally mutated the four differential amino acid residues located in fragment 2 of PcAS based on the protein alignment result of PcAS and PchAS (**Figure 4A** and **Supplementary Figure S3**). As shown in **Figure 4B**, mutant proteins (W260F, W260FH296D, W260FI337V, and W260FQ397P) produced one more product, lupeol, compared with the original PcAS. However, mutant proteins (H296D, H296DI337V, H296DQ390P, I337V, I337VQ390P, and Q390P) only produced the single product *β*-amyrin as PcAS (**Supplementary Figures S5A, B**). That is to say, either point or multiple mutations of 296, 337, and 397 in fragment 2 couldn′t change the function of PcAS (**Figure 4B** and **Supplementary Figure S5**). Only when the 260 tryptophan (W) was mutated to phenylalanine (F), the function of PcAS changed from the single product (*β*-amyrin) to multiple products (lupeol and *β*-amyrin). The 260th amino acid of PcAS and PchAS is the site leading to functional difference, which the 781st and 782nd nucleotides of *PcAS* and *PchAS* CDS are GG and TT, respectively.

### 2.5 The analysis of total triterpenoid content in dried radix of *Pulsatilla chinensis* and *Pulsatilla cernua*

**Figure 5:**
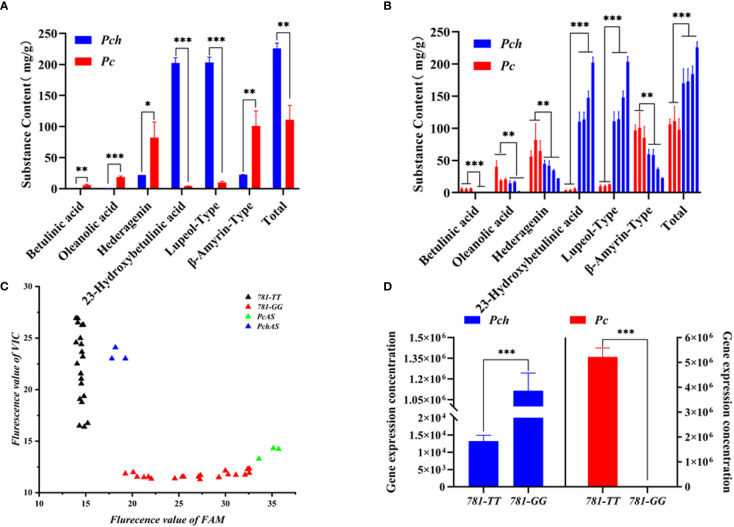
Triterpene acid content, *PcAS* and *PchAS* type gene expression in *Pulsatilla ceruna* and *P. chinensis*. **(A)** Different type triterpene acid contents in *P. ceruna* and *P. chinensis* freeze-drying roots, respectively; **(B)** Different type triterpene acid contents in *P. ceruna* and *P. chinensis* decoction pieces, respectively; **(C)** The FAM and VIC fluorescent value of qPCR which *P. ceruna*, *P. chinensis*, plasmid pET32a-*PcAS* and plasmid pET32a-*PchAS* as template; **(D)** Absolute gene expression of *PcAS* and *PchAS* type in *P. ceruna* and *P. chinensis*, 781-*TT* and 781-*GG* represent *PchAS* and *PcAS* type gene. Student’s t-test: *, *P* < 0.05; **, *P* < 0.01; ***, *P* < 0.001.

To investigate the types and contents of triterpenoid saponins in *P. chinensis* and *P. cernua*, we extracted triterpenoids from the roots of *P. chinensis* and *P. cernua* by two-phase hydrolysis method and absolutely quantified the content of triterpenoid by HPLC-UV. The results showed that the content of betulinic acid was lowest in triterpenoid acid, which ranged from 0.33 mg/g to 6.57 mg/g. Betulinic acid content in *P. ceruna* was higher than in *P. chinensis* (**Figures 5A, B**). Oleanolic acid content ranged from 0.46 mg/g to 40.22 mg/g, and the content of oleanolic acid in *P. ceruna* was higher than in *P. chinensis* (**Figures 5A, B**). It was higher than betulinic acid but lower than hederagenin and 23-hydroxybetulinic acid in every *Pulsatilla* material used in this study. The content of hederagenin in *P. ceruna* was higher than in *P. chinensis*, which ranged from 22.24 mg/g to 82.26 mg/g. The content of hederagenin was the highest triterpenoid in *P. ceruna*, while was lower than 23-hydroxybetulinic acid in *P. chinensis.* 23-Hydroxybetulinic acid was the dominant component in *P. chinensis*. The content of 23-hydroxybetulinic acid ranged from 3.52 mg/g to 202.47 mg/g. Total triterpenoids in *P. chinensis* (ranged from 170.35 mg/g to 226.05 mg) were higher than those in *P. cernua* (ranged from 98.07 mg/g to 111.11 mg/g) (**Figures 5A, B**). In addition, the contents of lupeol-type triterpenoids in *P. chinensis* were significantly higher than those of *β*-amyrin-type, while the contents of *β*-amyrin-type triterpenoids were significantly higher than the contents of lupeol-type triterpenoids in *P. cernua* (**Figures 5A, B**).

### 2.6 Absolute quantification of *PcAS* and *PchAS* genes expression in roots of *Pulsatilla chinensis* and *Pulsatilla cernua*

Due to the functional differences of the *AS* genes cloned from *P. chinensis* and *P. cernua*, we suspected that the difference of triterpenoid saponin types between *P. chinensis* and *P. cernua* were caused by the expression levels of the two gene types. Therefore, we analyzed the expression levels of *AS* gene type in *P. chinensis* and *P. cernua*. Since the site leading to functional difference is the 781st and 782nd nucleotides of *PcAS* and *PchAS* (GG/TT), so we used the TaqMan probe method to analyze the gene expression in different species. We designed a pair of specific primers (AS-Tq-F/R), and two probes for 781-*GG*-type and 781-*TT*-type. The probes added the MGB marker at 3*′* ends to improve the specificity and add FAM fluorescent signal for 781-*GG*-type and VIC fluorescent signal for 781-*TT*-type. We first collected these two fluorescent terminal signals of *P. chinensis* and *P. cernua* by terminal detection method. As shown in **Figure 5C**, these two different genotypes were expressed in both *P. chinensis* and *P. cernua*. Subsequently, the standard curves of these two genotypes were constructed respectively to absolutely quantitate their expression in *P. chinensis* and *P. cernua* (**Supplementary Figure S6**). As shown in **Figure 5D**, the expression of 781-*TT*-type was under the quantitative line and 781-*GG*-type gene expression was 5.11 × 10^6^ in *P. cernua*, while the expression of 781-*TT*-type gene expression level in *P. chinensis* (1.12 × 10^6^) was about 100 times higher than the 781-*GG* type (1.32 × 10^4^).

### 2.7 Gene copy number analysis of 781-*TT*-type and 781-*GG*-type in *Pulsatilla chinensis*, *Pulsatilla cernua* and *Pulsatilla turczaninovii*

**Figure 6:**
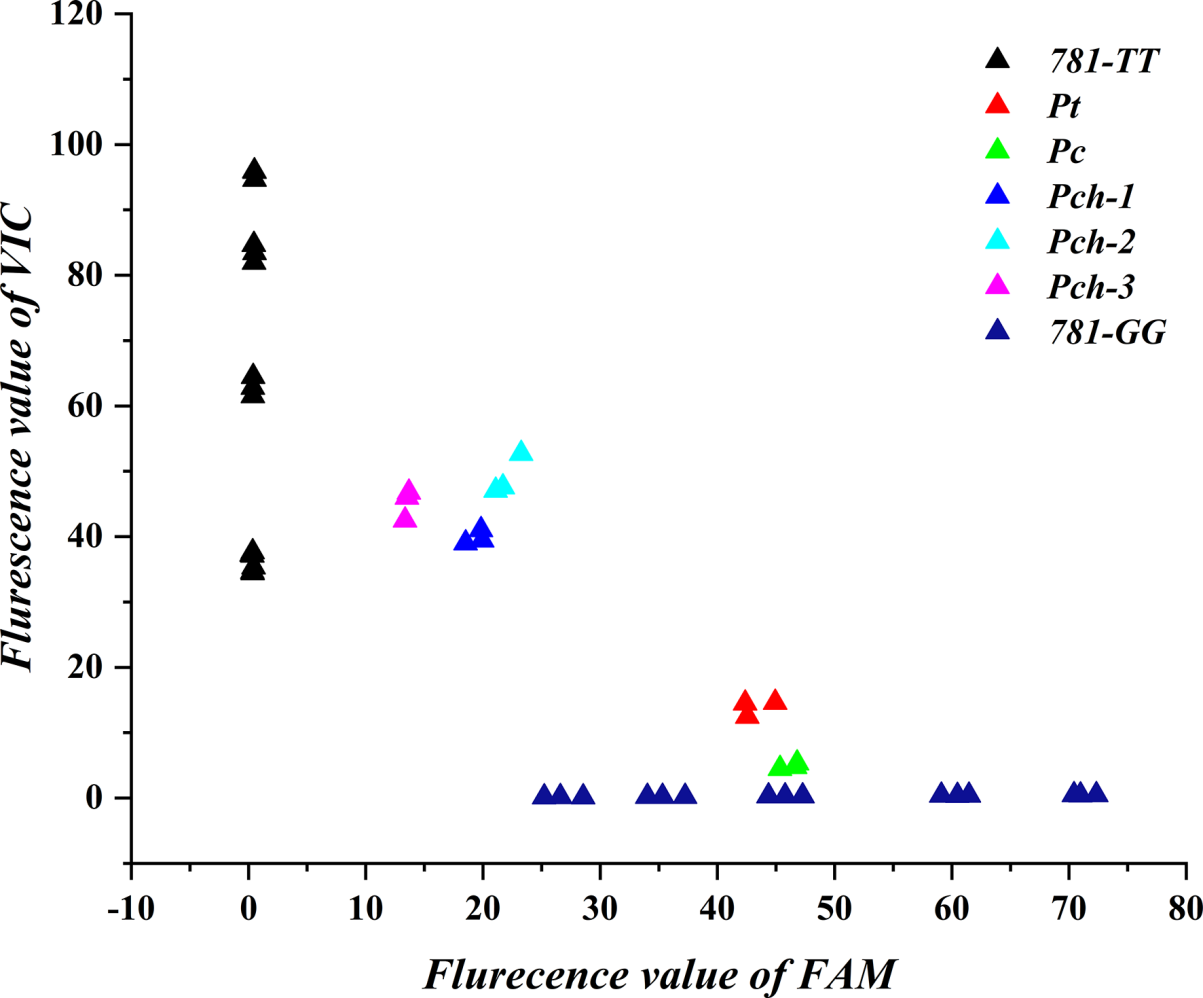
Gene copy number analysis of 781-*TT* and 781-*GG* in *Pulsatilla ceruna, P. chinensis* and *P. turczaninovii. Pc, Pch*, and *Pt* represent *P. ceruna, P. chinensis* and *P. turczaninovii*, respectively. 781-*TT* and 781-*GG* represent *PchAS* and *PcAS* type gene standard, respectively.

To explain the 781-*TT*-type and 781-*GG*-type gene expression difference in the *Pulsatilla* genus, we analyzed relative gene copy numbers of the 781-*GG*-type and 781-*TT*-type gene in *P. chinensis*, *P. cernua*, and *P. turczaninovii*. We first obtained and sequenced the full-length *AS* gene sequences from the DNA of these three *Pulsatilla* species by primer F4 and primer R4 listed in **Supplementary Table S1**. As shown in **Supplementary Figure S7**, the *AS* gene contains 12 introns and 13 extrons. Then we randomly choose four single clones from each species to sequence. The results indicated that there were three clones as 781-*TT*-type and one clone as 781-*GG*-type in *P. chinensis*, while all four clones were 781-*GG*-type in *P. cernua* and *P. turczaninovii*, respectively (**Supplementary Figure S8**). Finally, we analyzed these two genotypes by qPCR. By the method used to analyze the gene expression levels, we used the plasmids as the standard to quantitate the gene copy numbers. The primers were designed based on gene sequences of *PcAS* and *PchAS* (AS-TAQ-F/R). The probes used in this experiment were the 781-*GG* probe and the 781-*TT* probe. Then, we collected terminal signals of FAM and VIC fluorescent of *P. chinensis*, *P. cernua* and *P. turczaninovii*, respectively. As the results shown in **Figure 6**, three different *P. chinensis* materials have strong fluorescent signals of VIC and weak fluorescent signals of FAM, while the fluorescent signals of VIC and FAM in *P. cernua* and *P. turczaninovii* were opposite with in *P. chinensis.* This result suggests that in these three *Pulsatilla* species, the gene copy numbers of 781-*GG*-type and 781-*TT*-type are different. In *P. chinensis*, the 781-*TT*-type gene copy numbers were higher than the 781-*GG*-type. However, in *P. cernua* and *P. turczaninovii*, the 781-*TT*-type gene copy numbers were lower than the 781-*GG*-type.

## 3 Discussion

The research on the *Pulsatilla* genus mainly focused on the separation of compounds and the pharmacological activity analysis of the main active components. While up to May 2022, more than 100 triterpenoid saponins have been isolated from the *Pulsatilla* genus (Wang et al., 2020; Zha et al., 2020; Xu et al., 2022). These isolated compounds are mainly lupeol-type and oleanolic acid-type, and only one compound belongs to ursolic acid-type (Ding et al., 2010). However, the biosynthetic pathway of all separated compounds in *Pulsatilla* genus has not been analyzed until now, and there was little omics data to support us to reveal it. Therefore, we cloned two 2,3-oxidosqualene cyclase genes from *P. chinensis* and its closely related species *P. cerua* through homologous cloning. These two genes are similar to *Negro grass β*-amyrin synthase gene that has been functionally verified. According to the separate compounds and triterpenoid saponin contents in the *Pulsatilla* genus (Jin et al., 2018), we speculated that besides the *β*-amyrin syntheses, there should exist the lupeol synthases in the *Pulsatilla* genus, especially in *P. chinensis.* Because the content of anemoside B4, which is a kind of lupeol-type triterpene saponin, is rich in the roots of *P. chinensis* (Jiang et al., 2021). In order to verify the functions of *PcAS* and *PchAS*, we expressed these genes in 2,3-oxisqualene high-yield yeast strain ATCC4021900 and leaves of *N. benthamiana*. The results shown in **Figures 2C, D** indicate PcAS is a single functional enzyme that only produces *β*-amyrin, while PchAS is a multifunctional enzyme, and its main product is lupeol.

At present, about 170 OSC genes from plants have been functionally analyzed (Chen et al., 2021; Wang et al., 2021). At the same time, the researchers also carried out targeted mutation to find the key active sites of OSC genes (Souza-Moreira et al., 2016; Ito et al., 2017; Aiba et al., 2018; Suzuki et al., 2019). The key active sites affecting the products of the family members were mainly located in the N-terminal, MXCYC conservative region, and C-terminal (Guo et al., 2022). For example, in 1999, Kushiro et, al. found that about 20 residues from Cys260 to Trp340 determined the activity of *Panax ginseng β*-amyrin synthase and *Arabidopsis thaliana* lupeol synthase. In addition, F474 of *Euphorbia tirucalli β*-amyrin synthase has a key role in affording the correct folding of the substrate (Ito et al., 2014). While the *AS* gene from *P. chinensis* and *P. cernua* has 27 different amino acid residues in 759 amino acids (**Supplementary Figure S3**), and these 27 different amino acids are distributed in the whole gene region. Therefore, to determine the main functional diversity region, we fused these two *AS* gene sequences obtained from *P. chinensis* and *P. cernua*, as shown in **Figure 3A**. Functional analysis of the chimera proteins showed that 145-456 amino acids were important to these two AS functions.

There contains four different amino acid residues in fragment 145-456 between *P. chinensis* and *P. cernua*. To further identify the key site leading to its functional difference, we proceed with directional mutation on these different amino acid residues. The results showed that if the 260-position amino acid W of PcAS, which located in the MXCYCR domain, was mutant to F, its catalysate changed from the single product to multiple products. The combination mutation of this site with any other three sites maintained the change (**Figure 4**). However, when this site did not change, any single mutation or combination mutation at the other three sites will not change the catalysate type (**Supplementary Figure S5**). Therefore, the 260-position amino acid is considered as the main active site affecting PcAS and PchAS function difference. This result is consistent with previous studies. In 2000, to identify the amino acid residues responsible for *β*-amyrin (PNY) and lupeol (OEW) synthase product specificity, site-directed mutagenesis on these two synthases was carried out. In this experiment, the 259-position amino acid W of *β*-amyrin synthase was mutated to Leu, and the 259-position amino acid L of lupeol synthase was mutated to Trp. The catalysate analysis revealed that W259 of *β*-amyrin synthase and L259 of lupeol synthase were the key amino acid residues (Dang and Prestwich, 2000). Our results provide that besides L, when another key amino acid F exists in the domain of MXCYXR of AS, the catalysate is lupeol.

As the main active component of *P. chinensis*, anemoside B4 is a kind of lupeol-type triterpenoid saponin. However, the content of anemoside B4 in other species of the *Pulsatilla* genus cannot reach the stipulation of the Chinese Pharmacopoeia (4.6% dry weight). We speculate that this phenomenon may be due to the insufficient supply of lupeol in *P. cernua*. However, lupeol is mostly oxidized to form betulinic acid and 23-hydroxybetulinic acid, and *β*-amyrin is oxidized to form oleanolic acid and hederagenin in plants. Therefore, we used the two-phase extract method to assess extracted saponins and aglycones after acidolysis of *P. chinensis* and *P. cernua*. Various aglycones were separated and their contents were accurately quantified (**Figures 5A, B**). The results showed that lupeol-type triterpenoid saponins were the main products in *P. chinensis*, and their aglycones were mainly oxidized at 23-position, while oleanane-type triterpenoid saponins were main products in *P. cernua*.

Because the difference in the type of aglycone between *P. chinensis* and *P. cernua* is caused by 2,3-oxidoqualene cyclase, we believe that the difference in the contents of triterpenoid saponins between *P. chinensis* and *P. cernua* is caused by the different expression of the 781-*TT* and 781-*GG* genotypes. Therefore, we absolutely quantitated the expression of these two genotypes in *P. chinensis* and *P. cernua*. As shown in **Figures 5C, D**, these two genotypes were expressed in both *P. chinensis* and *P. cernua*, while the 781-*TT* type was mainly expressed in *P. chinensis* and the 781-*GG* type was mainly expressed in *P. cernua*. This result is consistent with the different type triterpenoid saponin content of *P. chinensis* and *P. cernua*. In other words, the expressions of these two genotypes influence the biosynthesis and accumulation of different type triterpenoid saponins in *P. chinensis* and *P. cernua*.

In order to further find out whether the expression differences of the 781-*GG* and 781-*TT* genotypes in *P. chinensis* and *P. cernua* were caused by the difference of the gene copy number, we analyzed the 781-*GG* and 781-*TT* genotype copy number in the genome of *P. chinensis*, *P. cernua* and *P. turczaninovii* (anemoside B4 content is under 4.6%) (Song et al., 2021) by the qPCR relative quantitative method. The results showed that 781-*GG* type copy number was more than the 781-*TT* type in *P. cernua*, and the 781*-TT* type copy number was more than the 781-*GG* type in *P. chinensis*.

To sum up, we obtained two OSC genes from *P. chinensis* and *P. cernua* through homologous cloning and verified their functions in yeast and *N. benthamiana*. Then we found out the key amino acid which leads to the function difference of PcAS and PchAS by gene fusion and site-directed mutagenesis. In addition, we clarified the reason for the triterpenoid type difference in *P. chinensis* and *P. cernua* by combined analysis of different triterpenoid content and gene expression of 781-*TT* and 781-*GG* type *AS*. At last, we explained that different gene expression of the 781-*TT* and 781-*GG* type *AS* in *P. chinensis* and *P. cernua* was caused by the difference of gene copy numbers. This study provided basic information for the molecular breeding of *P. chinensis*. In addition, our study provides a usable molecular mark to identify the *P. chinensis* decoction pieces.

## 4 Materials and methods

### 4.1 Plant materials

*Pulsatilla chinensis* and *P. cernua* plants used for gene expression and metabolite component analysis were obtained from Beijing and Liaoning, China, then planted at the Institute of Botany, Chinese Academy of Science for three months. *P.turczaninovii* seeds were obtained from the germplasm bank of wild species which were used for DNA extraction. After the seeds germinated in the petri dish with five water-soaking filter papers at 25°C, the seedlings were planted in a 20 cm circular pot with turfy soil, and cultured in the 16 h light/8 h night greenhouse at 23°C.

### 4.2 RNA and cDNA preparation

Plant total RNA was extracted from the roots by RNAprep pure plant kit (DP441, TIANGEN). 1 µg total RNA was used for first strand cDNA synthesis with HiScript^®^ III 1st Strand cDNA Synthesis Kit (+gDNA wiper) (R312, Vazyme). In addition, in order to obtain the *OSC* core sequence of *P. chinensis* and *P. cernua*, we first used the primers reported in previous studies (Guhling et al., 2006), and the sequences were listed in **Supplementary Table S1**. Undesirably, these primers were not suitable for *OSCs* of *P. chinensis* and *P. cernua*. Therefore, we designed primers based on highly conserved regions of OSC protein sequences deposited in GenBank used Condehop, which were degenerate at the 3*′* core region, and non-degenerate at the 5*′* consensus clamp region. The primer sequences were listed in **Supplementary Table S1**. The degenerate PCR primers F1&R1 and PrimeSTAR^®^ high-fidelity PCR enzyme (R045A, Takara) were used to obtain the core sequence of *OSCs* by touchdown PCR: 3 min 94°C, (10 s 98°C, 15 s 55°C, 30 s 72°C) × 10 cycles, (10 s 98°C, 5 s 55°C, 30 s 72°C) × 25 cycles, 5 min final extension at 72°C. The resulting PCR products were separated by 1% agarose gel electrophoresis and extracted using the V-ELUTE Gel Mini Purification Kit (ZPV202, ZOMANBIO). Then the purified products were recombined into the pLB vector (VT206, TIANGEN) and transformed into *Escherichia coli* TOP10 competent cells. The positive clones were sequenced by Sangon Biotech.

5*′* and 3*′* flanking fragment sequences of *OSC* were obtained by HiScript-TS 5*′*/3*′* RACE Kit (RA101, Vazyme). Firstly, 5*′* RACE-Ready cDNA and 3*′* RACE-Ready cDNA were synthesized. Then the 5*′* RACE-Ready cDNAs, 10 × Universal Primer Mix (UPM), 5*′* GSP Primer R2 were used to extend the 5*′* flanking fragment of *OSC*, and the 3*′* RACE-Ready cDNAs, 10 × Universal Primer Mix (UPM), 3*′* GSP Primer R2 for 3*′* flanking fragment. 5*′* GSP-Primer R2 and 3*′* GSP-Primer R2 were synthesized according to the obtained *OSC* core sequence (**Supplementary Table S1**). All RCR programs used in this stage were referenced in the kit instruction manual. The PCR products were purified, recombined, and finally transformed into *E. coli* TOP10 competent cells. The positive clones were sequenced by Sangon Biotech.

### 4.3 *OSC* full-length CDS sequence cloning and plasmids construction

Based on the *OSC* gene sequence obtained by RACE PCR from *P. chinensis*, Primers F4 and Primers R4 were used to obtain OSC CDS full-length sequence. These primers which 5*′* flanking sequences add 15 bp homologous sequences of destination vectors (pCAMBIA2300 for gene expression in plant, pET32a for gene expression analysis, and pESC-His for gene expression in yeast) were used to obtain the products with homologous sequences of vectors. Then the purified PCR products were homologously recombined into the linearized vectors by Exnase II (C112, Vazyme). Then the recombined vectors were transformed into *E. coli* TOP10 competent cells. The positive clones were sequenced by Sangon Biotech. In order to increase the substance of OSC, we constructed a vector (pSAK277-*NbHMGRa*) which can overexpress *N. benthamiana* 3-hydroxy-3-methylglutaryl reductase gene (*NbHMGRa*) in plants (Atsumi et al., 2018).

### 4.4 Functional characterization of OSCs in yeast

Plasmid pESC-His-*PcAS* or pESC-His-*PchAS* were transformed to an ERG7-deficient yeast strain (ATCC4021900) by LiAc/SS-DNA/PEG method (Gietz and Woods, 2002). For the expression of the galactose-inducible constructs, the positive colony was inoculated in 5 mL SD medium lacking uracil and supplemented with 2% glucose, and incubated overnight at 30°C and 220 rpm. Then 300 µL overnight cultures were inoculated into 30 mL of fresh SD medium lacking uracil and containing 2% glucose and grown at 30°C and 220 rpm in a 250 mL flask. After incubated for 24 h, the yeast cells were harvested by centrifugation (5 min, 5000 rpm), and washed twice with sterile water. At last, the cells were suspended at 30 mL SC-His medium with 2% galactose in a new 250 mL flask and incubated at 30°C and 220 rpm for 48 h. Then, the cells were harvested and refluxed with 10 mL 20% KOH/50% EtOH at 90°C for 1 h, then extracted three times with 10 mL of hexane. All hexane solutions were combined and evaporated under N_2_. Finally, the extractions were resuspended with 1 mL ethyl acetate, and stored at -20°C.

### 4.5 Functional characterization of OSCs in *Nicotiana benthamiana*

The plasmids pSAK277-*NbHMGR*, pCAMBIA2300, pCAMBIA2300-*PcAS*, and pCAMBIA2300-*PchAS* were individually transformed into *Agrobacterium tumefaciens* (strain GV3101) by the freeze-thaw method (Weigel and Glazebrook, 2006). The positive clones were incubated at 28°C, 200 rpm overnight in Luria-Bertani medium containing 50 mg/L kanamycin, 50 mg/L gentamicin, and 50 mg/L rifampicin. *A. tumefaciens* cells were washed with sterile water twice and resuspended with infiltration buffer (100 µM acetosyringone, 10 mM MgCl_2_ and 10 mM MES, pH 5.6). Adjust the OD_600_ of the suspension to 0.6 and staticly incubated at 28°C for 3 h. The suspensions of *A. tumefaciens* which harbor the plasmid pCAMBIA2300, pCAMBIA2300-*PcAS*, or pCAMBIA2300-*PchAS* were mixed with the pSAK277-*NbHMGR* in equal proportion and infiltrated into *N. benthamiana* leaves (three leaves per plant). Six days after agro-infiltration, the infiltrated leaves were harvested, frozen in liquid nitrogen immediately, and dried with the freeze-drying technology. 100 mg dried leaf powder was resuspended with 10 mL 20% KOH/50% EtOH solution and incubated at 90°C for 1h, then extracted with 10 mL hexane three times. All hexane solutions were evaporated with N_2_ and dissolved in 1 mL ethyl acetate. The extracts were stored at -20°C.

### 4.6 GC-MS analysis of metabolite compounds

Approximately 200 µL engineered yeast or *N. benthamiana* leaf extracts were evaporated with N_2_, then dissolved in pyridine and derivatized with *N*-methyl-*N*-(trimethylsilyl) trifluoroacetamide at 80°C for 30 min. The derivatized extracts were evaporated with N_2_ and dissolved in 200 µL hexane for gas chromatography and mass spectrometry (GC/MS) analysis. The GC-MS analysis was carried out as the previous study (Sandeep et al., 2019) with modified. For the GC-MS analysis, an aliquot (2 µL) was injected into the Shimadzu QP2010 gas chromatograph equipped with a WM-5MS capillary column (30 m × 250 µm, film thickness 0.25 µm) in unsplit mode. The injection temperature was 250°C, the carrier gas was helium with a flow rate of 1 mL/min the GC oven temperature was programmed from 180 to 300°C at 20°C min^−1^ and remained at 15 min. The ion trap temperature was 250°C. The electron energy was 70 eV. Spectra were recorded in the range of 50–750 m/z. All prominent peaks in the GC chromatogram were identified by comparison with a library database and the retention time of authentic standards.

### 4.7 Extraction and determination of total triterpenoid

#### 4.7.1 Extraction method

The diphasic solvent extraction method was used to analyze the contents of total triterpenoid compounds. First, 10 mL MeOH was added to 100 mg dry-root powder of *P. chinensis* and *P. cernua* and mixed for approximately 30 s. Ultrasonication was employed to assist and accelerate the extraction of triterpenoids in the ultrasonic bath (100W) at room temperature for 25 min. Then centrifuged at 4500 g for 10 min. The clear supernatant was evaporated to dryness with N_2_. The residues were resuspended in 1 mL methanol and transferred into a 100 mL flask. 15 mL 20% H_2_SO_4_ and 15 mL toluene were added to the flask. The mixture was incubated at 60°C, 150 rpm for 16 h. Then the organic phase was collected and evaporated with N_2_. The residues were resuspended in 500 µL MeOH and filtered through 0.22 µm filters for HPLC-UV analysis.

#### 4.7.2 HPLC-UV conditions

The chromatographic system consists of an HPLC (high-performance liquid chromatograph) and a UV detector. The stationary phase is a C30 column (250 x 4.6mm, Thermo Fisher Scientific). The column temperature was maintained at 40°C. The separation was performed by means of a linear gradient elution (phase A: water supplemented with 0.05% phosphoric acid, phase B: acetonitrile). The gradient was as follows: 55~60% B in 10 min, 60~80% B in 40 min, 95% B in 45 min, and 95% B for 5 min. The injected volume is 20 µL. All triterpene acids were detected at 210 nm.

### 4.8 Chimeric protein construction and site-directed mutagenesis

The homolog protein regions of PcAS and PchAS, which contain key sites influence OSC activity based on the previous study (Guo et al., 2022) were separated into three protein segments (**Figure 3A**). To generate chimeric proteins, the CDS segments of *PcAS* and *PchAS* were amplified by PCR using the primer pairs listed in **Supplementary Table S1** (AS-1381-F/R, AS-720-F/R, and AS-424-F/R). The segments were assembled and cloned into the vector pESC-His to obtain 6 chimeric proteins (**Figure 3A**).

After the key region of OSC was identified, the single and double site-directed mutations of PcAS were constructed by the Mut Express^®^IIFast Mutagenesis Kit (C214, Vazyme). The primer pairs used were listed in **Supplementary Table S1** (781-F/R, 892-F/R, 1013-F/R, and 1160-F/R).

### 4.9 Gene expression analysis

TaqMan-qPCR and absolute quantification were used to analyze the expression of *PcAS* and *PchAS* in different species. Firstly, the plasmids pET32a-*PcAS* and pET32a-*PchAS* were constructed as standards for these two gene types. And the gradient dilutions of plasmids pET32a-*PcAS* and pET32a-*PchAS* were used to build standard curves for these two gene types, respectively. Secondly, the primers were designed by Primer Express 3.0.1. The primers of *PcAS* (781-*GG*) were modified with *FAM* fluorescent labeling and *PchAS* (781-*TT*) were modified with *VIC* fluorescent labeling in their 5′ ends, respectively. MGB was added to their 3′ ends. The primer sequence informations were listed in **Supplementary Table S1**. Then ChamQ Geno-SNP Probe Master Mix (Q811, Vazyme) and Roche LightCycler 480 were used to analyze the absolute expression of *PcAS* and *PchAS*.

### 4.10 Gene copy number analysis of 781-*TT* type AS and 781-*GG* type AS in *Pulsatilla ceruna*, *Pulsatilla chinensis* and *Pulsatilla turczaninovii*

TaqMan-qPCR was used to analyze the gene copy number in *P. ceruna*, *P. chinensis* and *P. turczaninovii* as previous study (Maron et al., 2013) with modified. Firstly, genomic DNA was isolated from leaves of *P. ceruna*, *P. chinensis* and *P. turczaninovii* by FastPure^®^ Plant DNA Isolation Mini Kit (DC104, Vazyme). Then the primers (AS-Tq-F/R) and probes 781-*TT/GG* with *VIC* and *FAM* fluorescent labeling, respectively, were designed by Primer Express 3.0.1 (**Supplementary Table S1**). Finally, ChamQ Geno-SNP Probe Master Mix (Q811, Vazyme) and Roche LightCycler 480 were used to analyze the fluorescence of *VIC* and *FAM* to explain 781-*TT* and 781-*GG* type gene copy number. In this experiment, the standards of 781-*TT* and 781-*GG* were the same as those used in gene expression.

## Data availability statement

The original contributions presented in the study are included in the article/**Supplementary Material**. Further inquiries can be directed to the corresponding authors.

## Author contributions

JJ and AL designed the experiments and supervised the study. XL and JJ performed most of the experiments and wrote the manuscript. YX and JD performed the HPLC-UV analysis. All authors contributed to the article and approved the submitted version.

## Supplementary material

Supplementary Figure 1 Homologous cloning electrophoretogram of *AS* genes from *Pulsatilla ceruna* and *P. chinensis.* **(A)** The *AS* gene core fragment cloning from *P. ceruna* and *P. chinensis*; **(B)** 5′ and 3′ flank fragment of *P. chinensis AS* genes. **(C)** Full-length *AS* genes of *P. ceruna* and *P. chinensis.*

Supplementary Figure 2 Phylogenetic analysis of PcAS and PchAS with the OSCs of other species.

Supplementary Figure 3 Sequence alignment of AS proteins from *Nigella sativa (*NsAS*), Pulsatilla ceruna (*PcAS*)* and *Pulsatilla chinensis (*PchAS*)*.

Supplementary Figure 4 GC-MS analysis of *β*-amyrin and lupeol standard and the contents of PcAS and PchAS catalysate. The 218 and 189 ion counts of *β*-amyrin and lupeol are shown, respectively.

Supplementary Figure 5 Functional characterization of mutant protein of I337V, H296D, Q397P. **(A)** GC–MS analyses of the yeast extraction expressing the mutant proteins contain the I337V with/without other sites; **(B)** GC–MS analyses of H296D, Q397P and both these two sites mutant proteins expressed yeast extraction. The 218 and 189 ion counts of *β*-amyrin and lupeol are shown, respectively.

Supplementary Figure 6 Standard curve of 781*-TT* (*PchAS*) and 781*-GG* (*PcAS*) gene type.

Supplementary Figure 7 Sequence alignment of *AS* gene DNA full-length sequences and CDS full-length sequences.

Supplementary Figure 8 The *AS* gene core fragment sequences of *AS* genes cloned from the DNA of *Pulsatilla ceruna, P. chinensis* and *P. turczaninovii*.

Supplementary Table 1 Primers used in this article.
