# Supplementary figures and images for: The triterpenoid saponin content difference is associated with the two type oxidosqualene cyclase gene copy numbers of Pulsatilla chinensis and Pulsatilla cernua
Source: Front Plant Sci. 2023 Feb 23;14:1144738. doi: 10.3389/fpls.2023.1144738 (PMC9995806; doi:10.3389/fpls.2023.1144738)

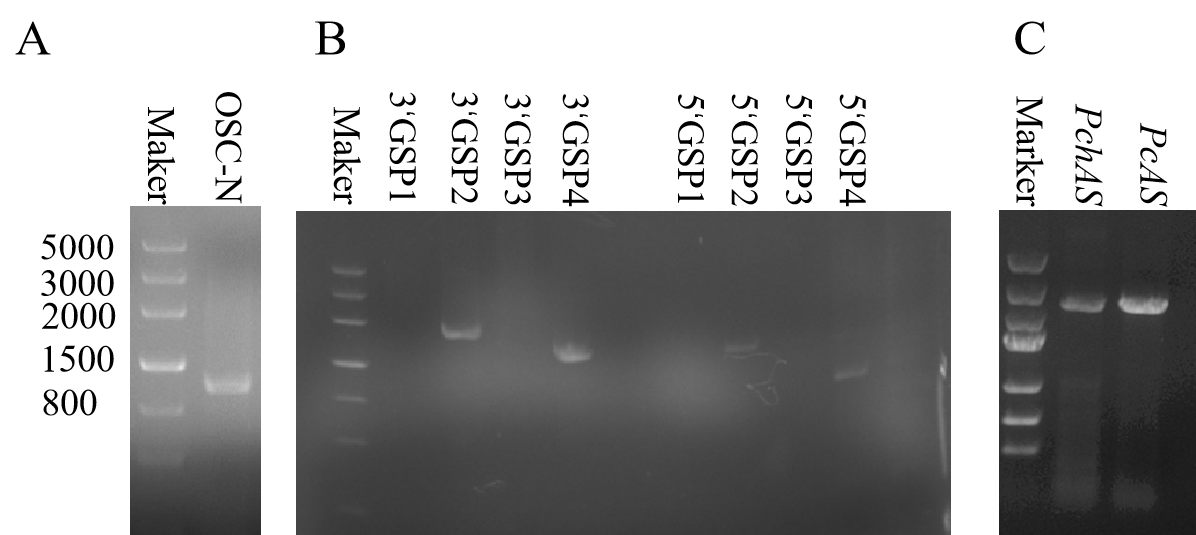

Supplement: Supplementary Figure 1 — Homologous cloning electrophoretogram of AS genes from Pulsatilla ceruna and P. chinensis. (A) The AS gene core fragment cloning from P. ceruna and P. chinensis; (B) 5′ and 3′ flank fragment of P. chinensis AS genes. (C) Full-length AS genes of P. ceruna and P. chinensis. [file Image_1.tif]

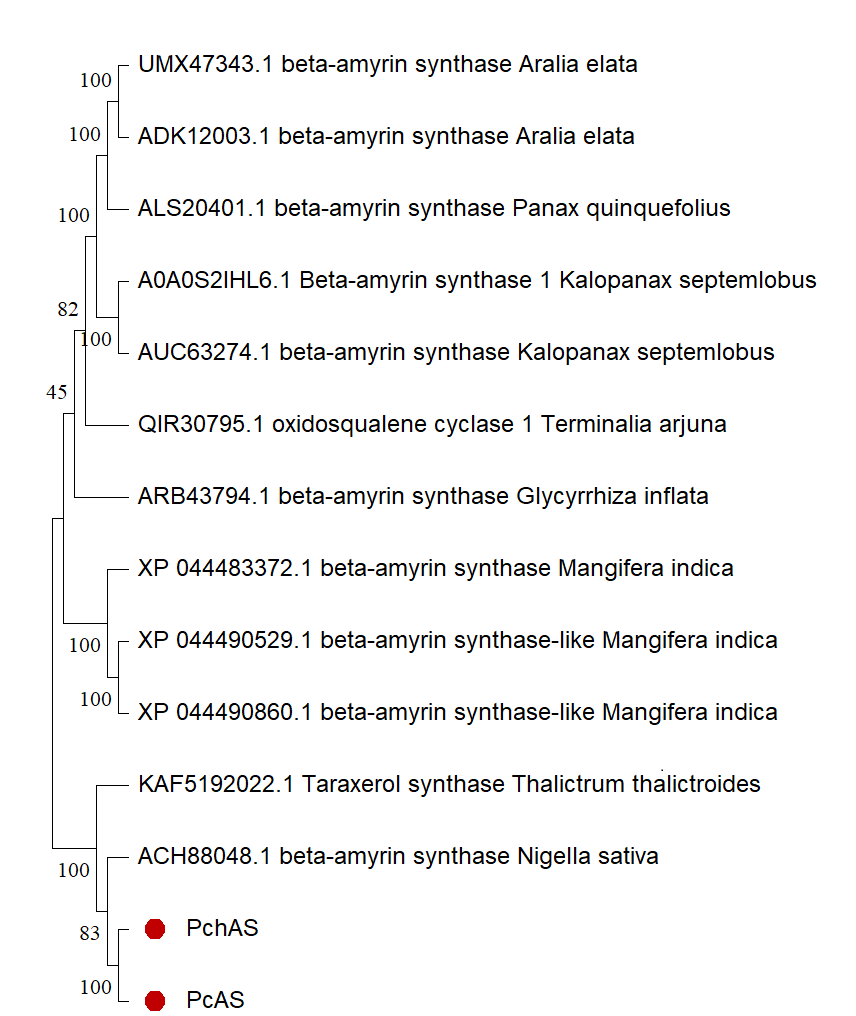

Supplement: Supplementary Figure 2 — Phylogenetic analysis of PcAS and PchAS with the OSCs of other species. [file Image_2.tif]

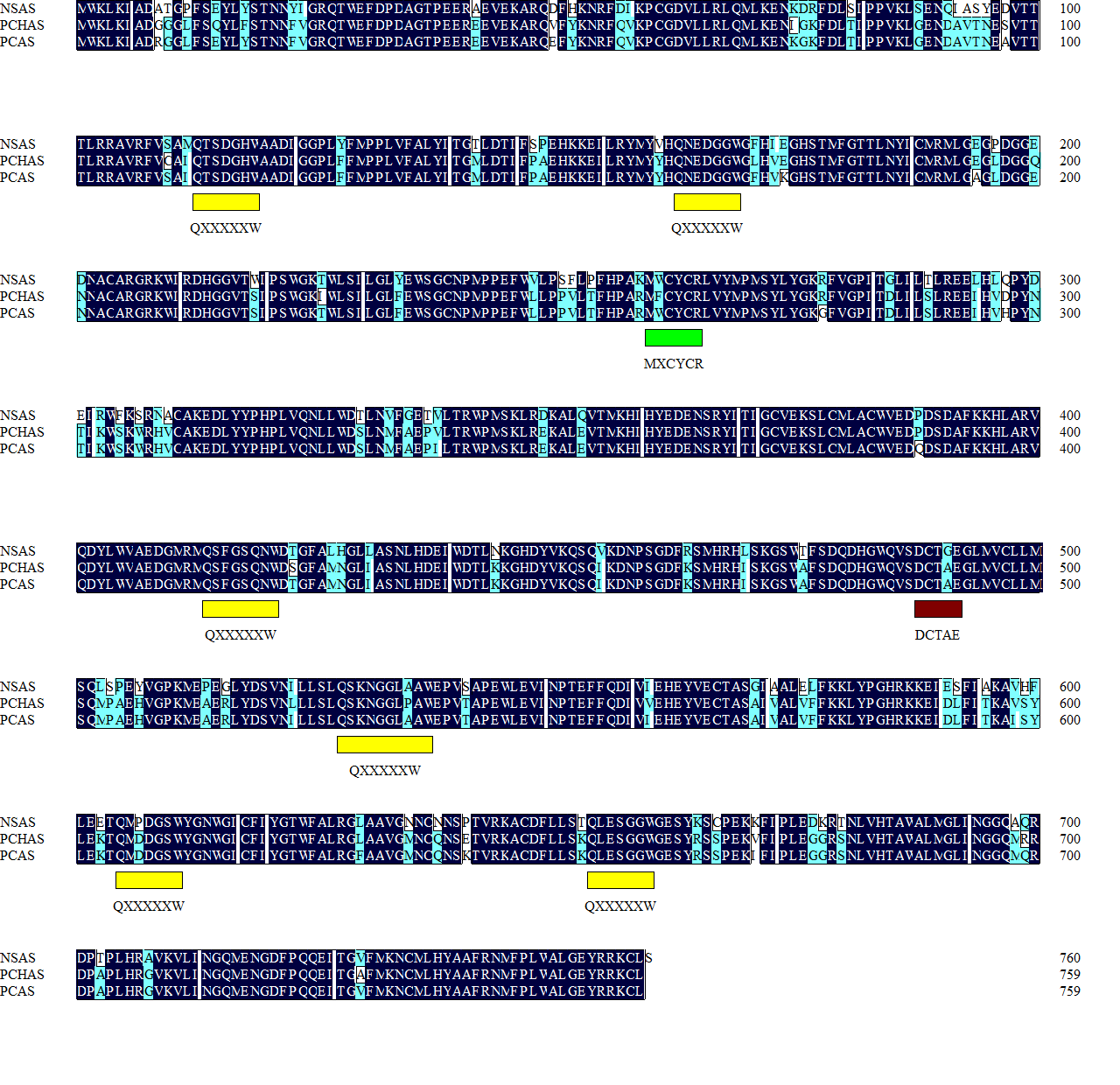

Supplement: Supplementary Figure 3 — Sequence alignment of AS proteins from Nigella sativa (NsAS), Pulsatilla ceruna (PcAS) and Pulsatilla chinensis (PchAS). [file Image_3.tif]

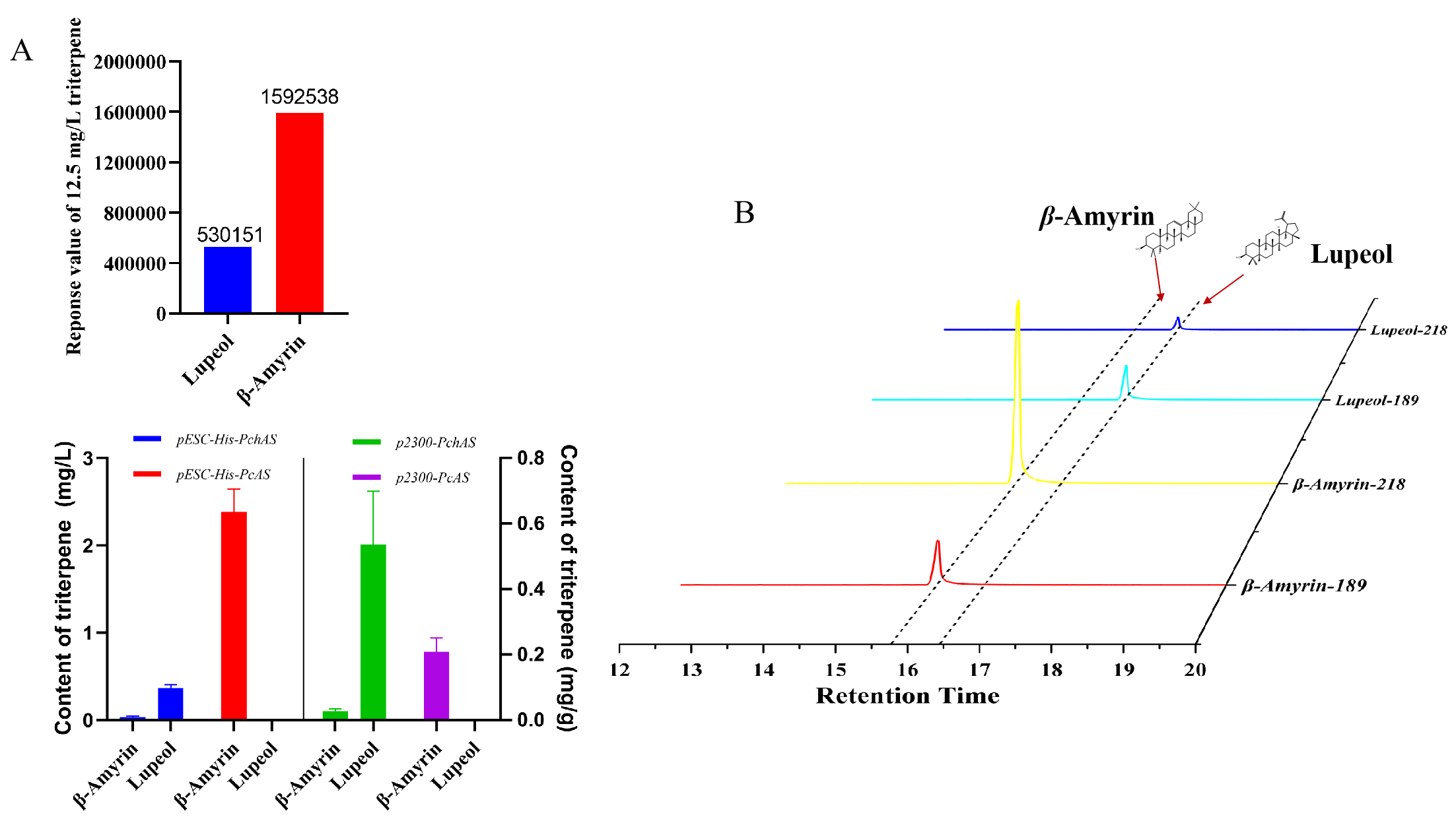

Supplement: Supplementary Figure 4 — GC-MS analysis of β-amyrin and lupeol standard and the contents of PcAS and PchAS catalysate. The 218 and 189 ion counts of β-amyrin and lupeol are shown, respectively. [file Image_4.tif]

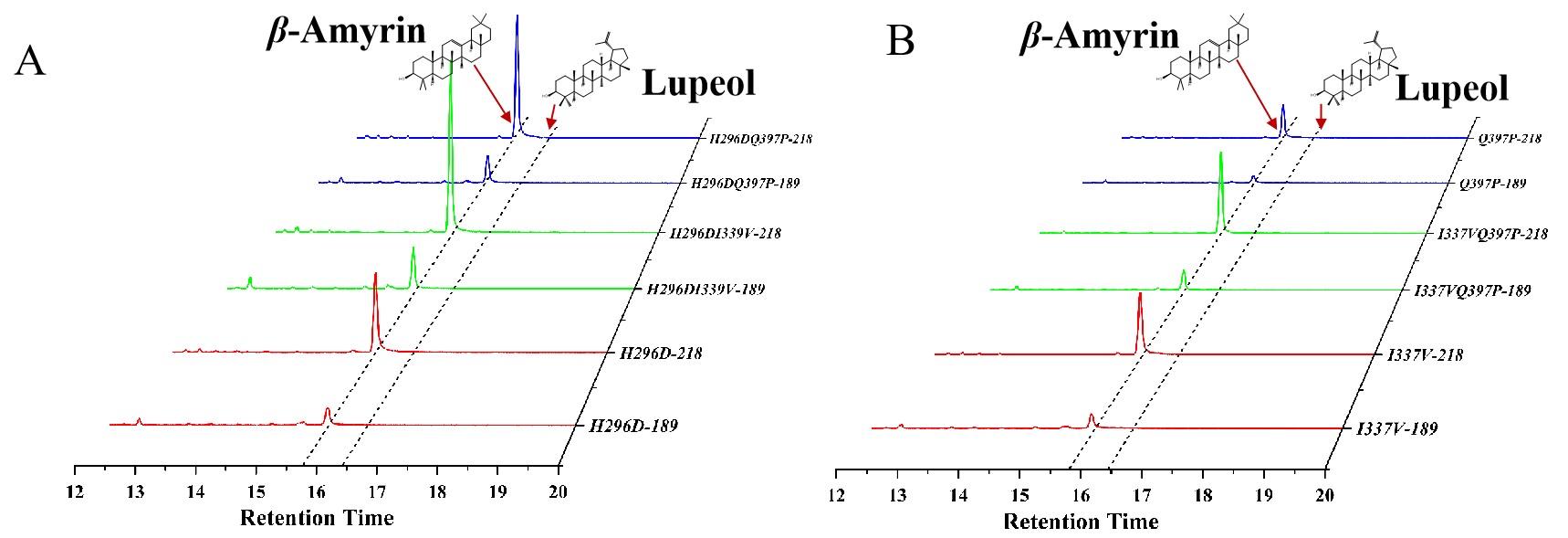

Supplement: Supplementary Figure 5 — Functional characterization of mutant protein of I337V, H296D, Q397P. (A) GC–MS analyses of the yeast extraction expressing the mutant proteins contain the I337V with/without other sites; (B) GC–MS analyses of H296D, Q397P and both these two sites mutant proteins expressed yeast extraction. The 218 and 189 ion counts of β-amyrin and lupeol are shown, respectively. [file Image_5.tif]

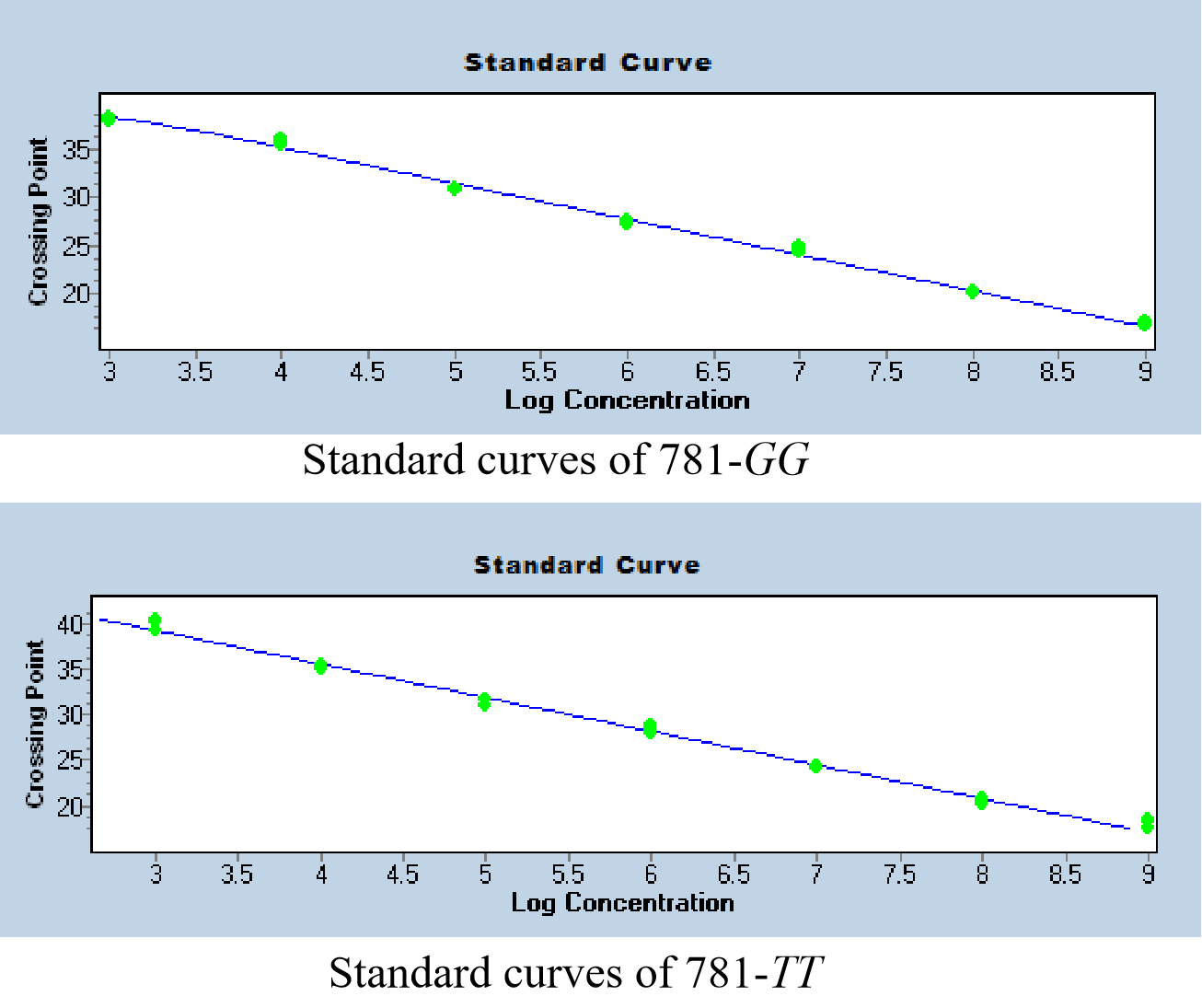

Supplement: Supplementary Figure 6 — Standard curve of 781-TT (PchAS) and 781-GG (PcAS) gene type. [file Image_6.tif]

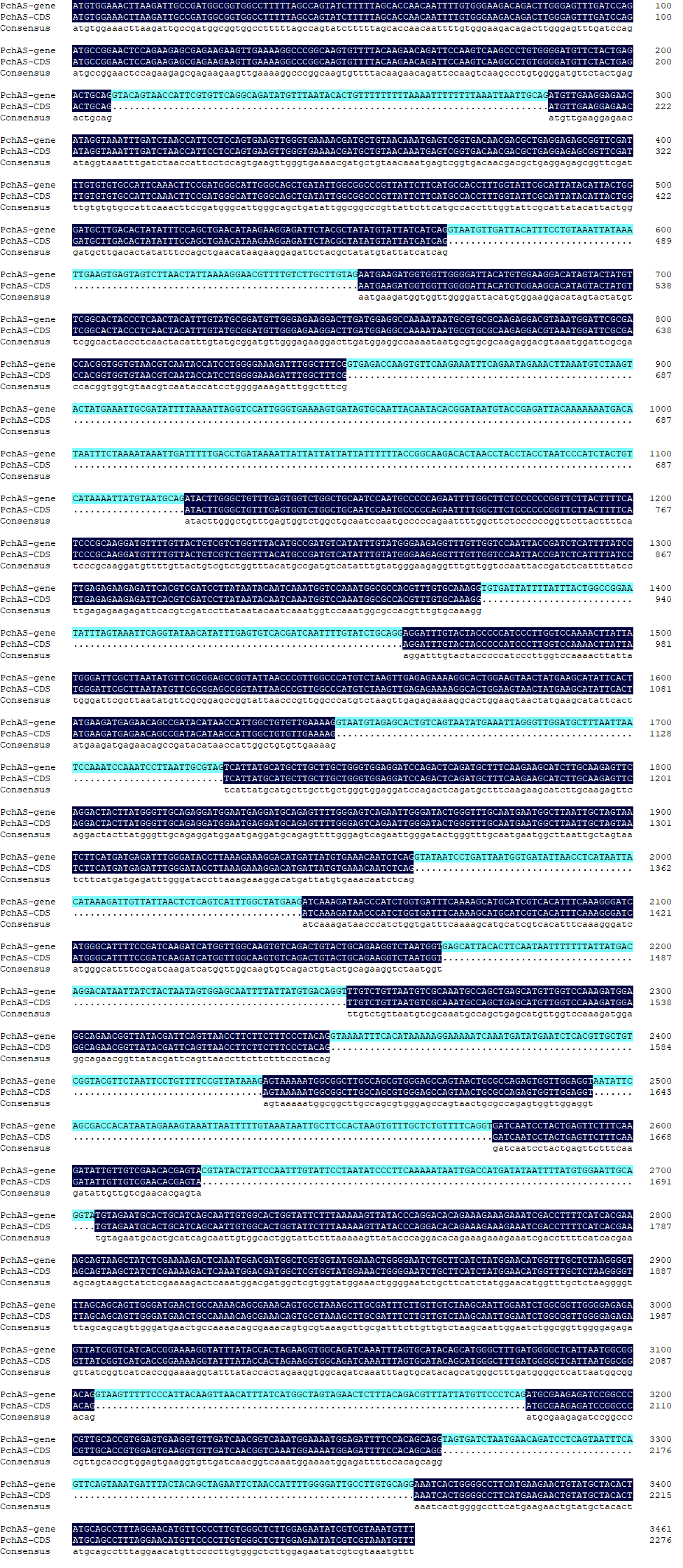

Supplement: Supplementary Figure 7 — Sequence alignment of AS gene DNA full-length sequences and CDS full-length sequences. [file Image_7.tif]

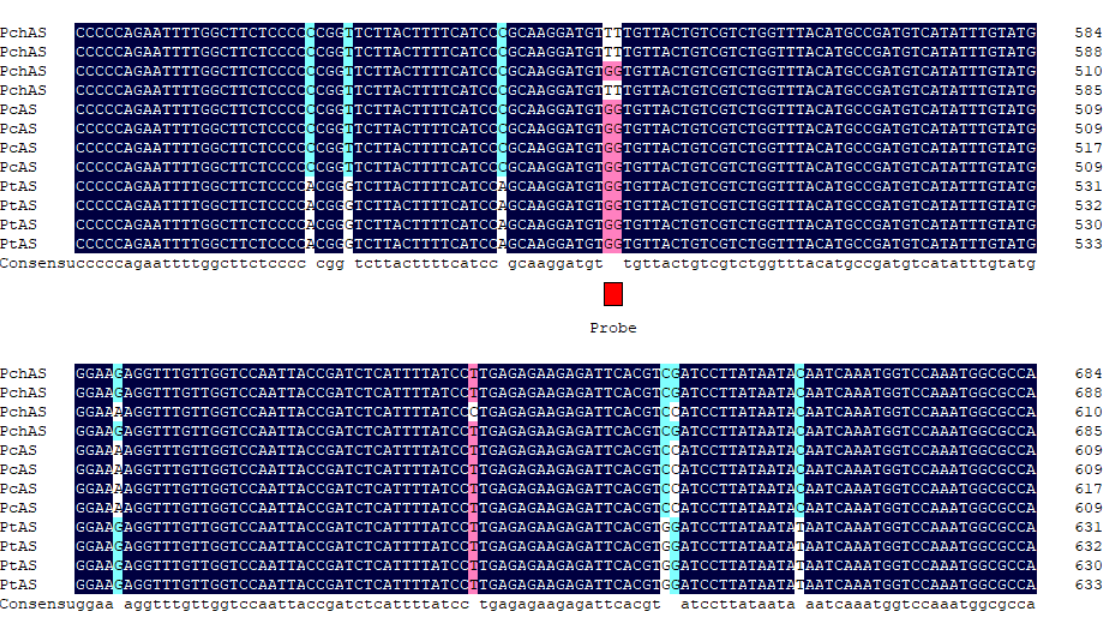

Supplement: Supplementary Figure 8 — The AS gene core fragment sequences of AS genes cloned from the DNA of Pulsatilla ceruna, P. chinensis and P. turczaninovii. [file Image_8.tif]
